# Two Cases of Post-Moderna COVID-19 Vaccine Encephalopathy Associated With Nonconvulsive Status Epilepticus

**DOI:** 10.7759/cureus.16172

**Published:** 2021-07-04

**Authors:** Benjamin D Liu, Corrado Ugolini, Pinky Jha

**Affiliations:** 1 Internal Medicine, Medical College of Wisconsin, Wawautosa, USA; 2 Internal Medicine, Medical College of Wisconsin, Wauwatosa, USA

**Keywords:** covid-19, covid-19-related encephalopathy, moderna vaccine encephalopathy, covid-19 vaccine complication, vaccine seizures, non-convulsive status epilepticus

## Abstract

Coronavirus disease 2019 (COVID-19) infections can cause many complications, including central nervous system (CNS) complications. One of the most common COVID-19 CNS complications is COVID-19 encephalopathy, a disorder characterized by cognitive impairment, altered consciousness, and even seizures. The Moderna COVID-19 vaccine is a recently approved mRNA-based vaccine aimed at preventing COVID-19 infections and their complications. Here, we describe two patients with no known neurological or psychiatric history who presented with encephalopathy and seizures that began after part one of their Moderna COVID-19 vaccine series. To our knowledge, there are no other reports of post-Moderna COVID-19 vaccine encephalopathy in the literature. We suggest a mechanism for this complication and thoroughly discuss why the Moderna vaccine is possibly responsible, by addressing confounders in our patients.

## Introduction

The coronavirus disease 2019 (COVID-19) is an intriguing respiratory infection that presents with a myriad of symptoms. Increasingly, reports of post-COVID-19 delirium, prolonged cognitive impairment, and even seizures are emerging, demonstrating a likely neurotropic component of the virus [1]. To prevent this and other severe or fatal complications, the Moderna COVID-19 vaccine was developed. This “Operation Warp speed” vaccine was approved on December 18, 2020, via emergency use authorization by the U.S. Food and Drug Administration after just under a year of development. This mRNA vaccine has an unprecedented 94.1% efficacy in preventing COVID-19 infections among adult participants in phase 3 clinical trials [2]. The side effect profile reported during the clinical trials was relatively benign, with most reactions being mild local reactions, fatigue, or headache. However, phase 3 clinical trials can never capture the entire profile of side effects that may occur once distributed to the public. A recent report of 23 deaths shortly after Pfizer COVID-19 vaccination in elderly patients has raised questions regarding whether this group may have unforeseen adverse outcomes in response to COVID-19 vaccination [3].

## Case presentation

Case 1

An 86-year-old woman with a history of diastolic dysfunction, chronic kidney disease stage 3, glaucoma, cataracts, and Type 2 diabetes mellitus was hospitalized 7 days after receiving her first Moderna COVID-19 vaccine administration. Her son reported that she was at baseline neurological function until she suddenly developed acute confusion with visual hallucinations and left frontal headache two days before admission. Notably, when symptoms first developed, her son brought her to the emergency department where a urinary tract infection was diagnosed, Cephalexin was prescribed, and she was discharged later that day. The patient was compliant with her antibiotics, with the help of her son, but continued to worsen in mental status. Per chart review, there was no evidence of cognitive dysfunction at baseline at her annual Medicare visit three months prior, and no known neurological or psychiatric history. The patient had also never used tobacco, alcohol, nor any other illicit drugs. There were no new medications except for topical prednisolone for recent left eye phacoemulsification and lens implant and the cephalexin.

On admission, she was afebrile, had a pulse of 61, respiratory rate of 20, saturating 99% on room air with a blood pressure of 150-170 over 66-75. There were no focal neurological deficits (FND) noted. However, physical exam was significant for word-finding difficulties and slow mentation. No suprapubic, abdominal nor costovertebral pain was noted. Laboratory studies were significant for erythrocyte sedimentation rate (ESR) of 107 mm/hr, C-reactive protein (CRP) of 1.2 mg/dL which elevated to 4.0 mg/dL on discharge, procalcitonin of 0.14 ng/mL and a blood glucose of 394 mg/dL with an osmolality of 308 mOsm/kg. Diabetes management was consulted with good management of blood glucose during admission. TSH, B1, complete metabolic panel, and folate were otherwise within normal limits (WNL). Venous blood gas and chest X-ray was unremarkable. B12, however, was elevated above 2,000 pg/mL. Respiratory infectious panel, including COVID-19, was negative. Urinalysis, as well as urine, blood, and CSF cultures, were negative as was the rapid plasma reagin (RPR) screen. Nevertheless, IV Ceftriaxone was started while inpatient due to the recent urinary tract infection.

CSF studies, including meningitis/encephalitis panel NAAT, oligoclonal bands, and Lyme antibody, were negative except for West Nile virus IgG but no IgM antibodies with minimal protein elevation. CT head without contrast and MRI brain with and without contrast showed no acute findings. Neurology, ophthalmology, and rheumatology were consulted. There was no evidence of giant cell arteritis. Two days into admission, continuous EEGs demonstrated non-convulsive focal status epilepticus treated with lorazepam and fosphenytoin. The patient’s seizures resolved, her neurological status significantly improved, and she was discharged home on day 6 of hospitalization with levetiracetam.

One month after hospitalization, the patient was seen in an outpatient neurology clinic with baseline neurologic status. She was compliant with her medication and had an age-appropriate neurological exam. She reported no family history of seizures at this visit. She also reported she had gotten her second Moderna COVID-19 vaccination two weeks after hospitalization with only local injection soreness.

Case 2

A 73-year-old man with a history of Crohn’s, hereditary hemochromatosis, hypertension, and hyperlipidemia was hospitalized 21 days after receiving his first Moderna COVID-19 vaccine. He was recently hospitalized for 2 days following 10 days of staring episodes, restlessness, and cognitive deficits that started 7 days after his Moderna COVID-19 vaccine. His wife described symptoms first starting as frequent episodes of staring and unresponsiveness for hours at an unknown frequency. The patient had no known neurological history nor any psychiatric history. Chart review revealed that there were no deficits on the Medicare annual wellness cognitive screen 7 months prior. The patient had never used tobacco, drinks 3-4 drinks per week and does not use any other illicit drugs. There were no new medications except for a prednisone 40mg taper that was started 5 weeks ago for a Crohn’s flare. Prednisone was discontinued at the prior 2-day hospitalization and the patient was discharged in improved condition following IV fluid administration and stable admission course, at patient and wife’s request. While at home, he developed hallucinations, worsening confusion and continued to have periods of unresponsiveness.

On admission 2 days later, the patient was afebrile, had a pulse of 90, respiratory rate 18 saturating 96% on room air with a blood pressure of 110-130 over 65-70. The initial physical exam was significant for word-finding difficulties and slow mentation but no FND and a benign abdominal exam. Laboratory studies were unremarkable except for a mild AKI. CRP, procalcitonin, and ESR were WNL. TSH, B12, B1, B9, complete blood count, liver function tests, ammonia, and morning cortisol were unrevealing. Respiratory infectious panel, including COVID-19, was negative. Urine and CSF cultures were negative as were HIV and RPR testing. Blood cultures were initially positive with Staphylococcus epidermidis and Staphylococcus hominis, and the patient was treated empirically for 3 days with IV ceftriaxone. However, these cultures were later thought to be contaminated following ID consultation due to negative repeat cultures, normal transthoracic echocardiogram, and normal chest x-ray. CSF studies, including meningitis/encephalitis panel Nucleic Acid Amplification Tests (NAAT), autoimmune encephalitis, and toxoplasma, were negative except for mildly elevated protein and glucose.

CT head and MRI brain showed no acute findings. The patient’s Saint Louis University Mental Status Examination (SLUMS) revealed 0/5 on delayed recall after 3 minutes, with a total score of 9/30 indicating severe deficits. Three days into admission, continuous EEG demonstrated non-convulsive status epilepticus, which was treated with lorazepam and levetiracetam loading and maintenance. The patient’s seizures resolved, neurological status significantly improved, and he was discharged home on day 8 of hospitalization.

Two months after hospitalization, the patient was seen remotely in outpatient neurology clinic. The patient and wife stated that they felt he was at baseline neurological status and were compliant with prescribed medications. The patient was able to recall 3 out of 3 words both immediately and after 5 minutes, although a true SLUMS test was not able to be conducted. The patient otherwise had a normal neurological exam for his age given what could be conducted remotely. The patient also provided further history at this time of never having symptoms worrisome for seizures or partial seizures, having no risk factors for epilepsy, and having no family history of seizures. After a thorough discussion, he eventually received his second Moderna COVID-19 vaccination a week after the clinic visit with no reported complications.

## Discussion

Acute encephalopathy and seizures have a wide basket of possible etiologies. Nevertheless, seizures following vaccinations have been noted as a possible complication [4]. At the initial submission of this article, there were no known reports of possible COVID-19 vaccination-related seizures. However, since then, one report has surfaced of a 68-year-old rural Indian man experiencing non-epileptic seizures four days after receiving his first dose of Covishield vaccine [5]. We are therefore the second report, to our knowledge, suggesting this possibility. 

Within our patient cohort, both elderly patients had no known neurological nor psychiatric history and began developing symptoms about a week after part one of their Moderna COVID-19 vaccinations. Our second patient was fortunate to get an extensive seizure history taken at his outpatient neurology visit, where it was noted that from birth, there were never any symptoms nor risk factors for epilepsy. To suddenly develop primary epilepsy at the age of 73 would therefore be extremely unlikely. In the acute inpatient setting, both of our patients had an extensive altered mental status workup. This included structural, metabolic, and infectious causes - all of which were not satisfactorily explanatory. Due to concern that these events may have been related to the Moderna COVID-19 vaccination, the primary medicine teams reported the events to the hospital's internal safety office who then reported to the federal level. 

One possible mechanism for the Moderna vaccine causing seizures and encephalopathy may be related to COVID-19 encephalopathy. It has been proposed that this common COVID-19 related CNS complication is caused by a cytokine storm triggered by the virus itself [6]. Severe acute respiratory syndrome coronavirus 2 (SARS-COV-2) has a spike glycoprotein that can bind the angiotensin-converting enzyme 2 (ACE-2) receptor. ACE2 binding and subsequent inhibition has been hypothesized to decrease brain-derived neurotrophic factor (BDNF). This theoretically reduces BDNF's anti-inflammatory action on neurons and its ability to attenuate microglial activation [7, 8]. Increased inflammatory signals can increase the likelihood of seizures, which is likely why there is an increased prevalence of seizures in intensive care unit COVID-19 patients [9, 10].

While the Moderna COVID-19 vaccine does not directly contain any proteins as an mRNA vaccine, it does encode for antigen S-2P [11]. This antigen includes the SARS-CoV-2 glycoprotein. As a result, we propose that the production of the spike protein, from cells translating the mRNA, triggers the same inflammatory cascade as a COVID-19 infection, resulting in these neurotropic effects. Notably, patient one fits this criterion with elevated inflammatory markers, CRP, ESR, and procalcitonin, approximately two days after symptoms first began, and 7 days after the first vaccination. Patient two, however, does not fit said criteria. It should be noted, however, that this patient’s inflammatory markers were only measured 21 days after the Moderna vaccination and 14 days after symptoms began. They were not measured during his previous 2-day hospitalization at an outside hospital. Curiously, the patient was on prednisone until 4 days before inflammatory markers were measured which should theoretically attenuate the response. As a result, we acknowledge that further research is needed to investigate this possibility.

We also do note that the primary possible confounders in this scenario are the presence of a urinary tract infection in patient one, the presence of steroid use in both patients, and the lack of a urine drug screen in both patients. While patient one did have a urinary tract infection, she was compliant with her antibiotics and was continued with ceftriaxone while inpatient. While urinary tract infections have been noted to cause acute encephalopathy in the elderly and febrile seizures, most commonly in infants, this patient remained afebrile, her vital signs remained stable and appropriate, and she had no suprapubic, abdominal nor costovertebral discomfort to suggest a complicated urinary tract infection [12]. Finally, urinalysis and cultures were negative on admission, and seizures were noted on the 4th day after antibiotic initiation.

Although both patients were using prescribed steroids when symptoms first began, patient one was using ophthalmic drops which are highly unlikely to cause such systemic manifestations. Patient two was nearing the end of his steroid taper and had not developed symptoms on the 50mg prednisone bursts. To suddenly develop symptoms five weeks later, on a much lower dose, is unlikely. Furthermore, five days after discontinuing prednisone, seizures were present on continuous EEG, and morning cortisol was within normal limits.

Finally, despite the lack of a urine drug screen, both patients denied any illicit drug use, smoking, or heavy drinking. Neither patients displayed typical symptoms of alcohol withdrawal, cocaine, methamphetamine, or any other illicit drug use known to cause seizures, considering the stable vital signs throughout their admission and lack of any suggestive physical exam findings [13, 14]. Furthermore, the duration of symptoms is inconsistent with seizures and altered mental status from acute intoxication or withdrawal from illicit drugs.

## Conclusions

We report two cases of seizures and encephalopathy after Moderna COVID-19 vaccination in two patients without any neurological or psychiatric history. Both patients had delirium and non-convulsive status epilepticus in the setting of an extensive negative workup. We recognize that attributing the pathology directly to the vaccine cannot be done in a simple case report or case series. Even examining large collections of possible adverse events, such as via the Vaccine Adverse Event Reporting System (VAERS) can only report associations and not causation. Nevertheless, we hope these findings create awareness of the possibility of an adverse Moderna COVID-19 vaccine-related event and for others to report similar cases should they arise. 
